# Social determinants of health and seasonal influenza vaccination in adults ≥65 years: a systematic review of qualitative and quantitative data

**DOI:** 10.1186/1471-2458-13-388

**Published:** 2013-04-25

**Authors:** Jason M Nagata, Isabel Hernández-Ramos, Anand Sivasankara Kurup, Daniel Albrecht, Claudia Vivas-Torrealba, Carlos Franco-Paredes

**Affiliations:** 1 School of Medicine, University of California, San Francisco, San Francisco, California, USA; 2 Microbiology Laboratory, School of Medicine, University of Guanajuato, Guanajuato, Mexico; 3 Social Determinants of Health, Department of Ethics and Social Determinants, World Health Organization, 20 Avenue Appia, Geneva, CH-1211, Switzerland; 4 Initiative for Vaccine Research, Department of Immunizations, Vaccine and Biologicals, World Health Organization, Geneva, Switzerland; 5 Hospital Infantil de México, Federico Gómez, Mexico City, Mexico; 6 Phoebe Memorial Hospital, Albany, Georgia, USA

**Keywords:** Influenza, Vaccination, Immunization, Elderly, Social determinants of health, Review, Qualitative synthesis, Thematic analysis

## Abstract

**Background:**

Vaccination against influenza is considered the most important public health intervention to prevent unnecessary hospitalizations and premature deaths related to influenza in the elderly, though there are significant inequities among global influenza vaccine resources, capacities, and policies. The objective of this study was to assess the social determinants of health preventing adults ≥65 years old from accessing and accepting seasonal influenza vaccination.

**Methods:**

A systematic search was performed in January 2011 using MEDLINE, ISI – Web of Science, PsycINFO, and CINAHL (1980–2011). Reference lists of articles were also examined. Selection criteria included qualitative and quantitative studies written in English that examined social determinants of and barriers against seasonal influenza vaccination among adults≥65 years. Two authors performed the quality assessment and data extraction. Thematic analysis was the main approach for joint synthesis, using identification and juxtaposition of themes associated with vaccination.

**Results:**

Overall, 58 studies were analyzed. Structural social determinants such as age, gender, marital status, education, ethnicity, socio-economic status, social and cultural values, as well as intermediary determinants including housing-place of residence, behavioral beliefs, social influences, previous vaccine experiences, perceived susceptibility, sources of information, and perceived health status influenced seasonal influenza vaccination. Healthcare system related factors including accessibility, affordability, knowledge and attitudes about vaccination, and physicians’ advice were also important determinants of vaccination.

**Conclusions:**

Our results demonstrate that the ability of adults ≥65 years to receive seasonal influenza vaccine is influenced by structural, intermediate, and healthcare-related social determinants which have an impact at the health system, provider, and individual levels.

## Background

The global burden of inter-pandemic influenza is estimated at 1 billion cases of flu, 3–5 million cases of severe illness, and 300,000-500,000 deaths annually [1], with about 90% of all influenza-related deaths occurring in adults aged 65 years or more and well-defined risk groups [2,3]. Influenza is an important contributor to the annual increase in hospitalizations and deaths attributed to pneumonia and influenza that is observed during the winter months, particularly among those ≥65 years old or those with chronic medical conditions including pulmonary, cardiovascular, or renal diseases as well as immunosuppression [3]. The primary goal of influenza vaccination in these high risk groups is to prevent unnecessary hospitalizations and premature deaths related to influenza, since episodes of influenza tend to exacerbate chronic medical conditions and lead to the occurrence of secondary bacterial pneumonias.

In the general population, immunization against influenza is considered the most important public health intervention to control seasonal, epidemic, and pandemic influenza [3-5]. Priority approaches and strategies to respond to an influenza pandemic are to achieve appropriate rates of vaccine uptake [6]. This would increase seasonal vaccine demand to stimulate market forces and augment supply, thus expanding the production capacity in a sustainable way [4,5]. There are, however, marked differences among countries’ capacities, priorities, and resources to establish influenza vaccination policies and strategies [7-10].

There have been previous reports, reviews [11], and a recent Cochrane systematic review [12] to assess the effectiveness of vaccines in preventing influenza, influenza-like illness, hospital admissions, and mortality in the elderly. For community dwelling elderly, the adjusted analyses from cohort and case control studies in the Cochrane review show that the effectiveness of the vaccine is modest, with reductions in the risk of hospitalizations for influenza or pneumonia, for respiratory or cardiac diseases, and for all-cause mortality (cohort studies) or death specifically from influenza and pneumonia (case–control studies) [12].

Other studies have tried to identify determinants of seasonal influenza vaccination [13], but few have focused on identifying social determinants within a framework of health equity [14] or their focus has been in other age groups [15]. Social determinants of health (SDH) play a critical role in disease occurrence, distribution, and consequences.

**Figure 1:**
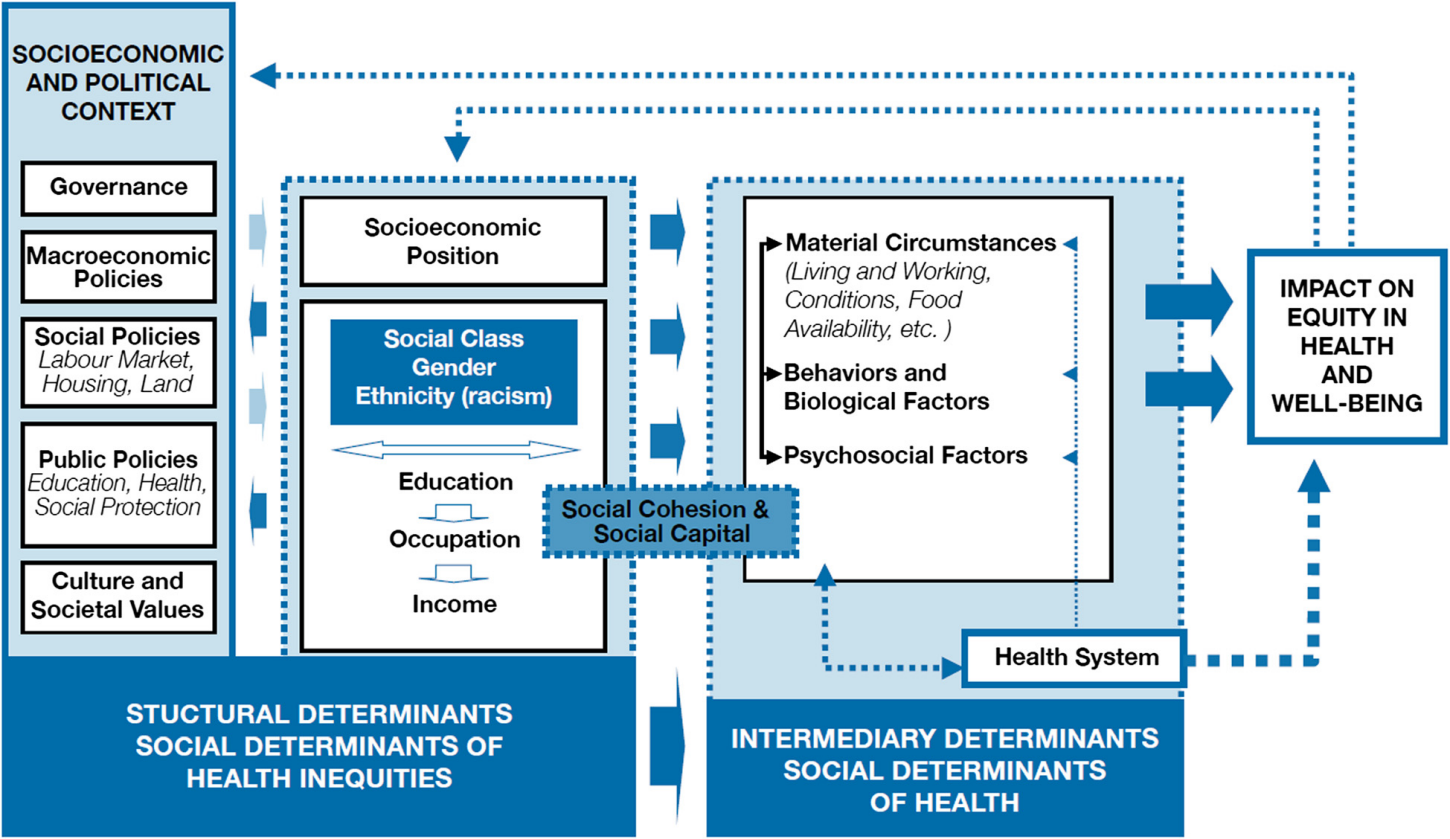
Commission on Social Determinants of Health conceptual framework (Solar & Irwin, 2010 [17]).

A Cochrane review about interventions to increase influenza vaccination rates of the elderly in the community found (with evidence from randomized controlled trials in developed countries) that personalized postcards and phone calls are effective, home visits and facilitators may be effective, but reminders to physicians are not [16]. This review did not include randomized controlled trials of society-level interventions or qualitative studies. To improve the understanding of the multidimensional challenge of yearly seasonal influenza vaccination among the elderly, the aim of this review was to assess the barriers that prevent elderly people from accessing and accepting seasonal influenza vaccine and its related social determinants. We used the conceptual framework that the Commission on Social Determinants of Health (CSDH) developed to identify determinants linked to seasonal influenza vaccination. This framework is based on a social production of disease approach, in which individual health outcomes and diseases and their unequal distribution across population groups are the result of the interaction of several determinants operating at different domains [17,18] (Figure 1). This framework contains three types of determinants: 1) Structural determinants; 2) Intermediate determinants; and 3) Determinants associated with the healthcare system.

## Methods

In this systematic review we considered qualitative and quantitative studies on seasonal influenza vaccination and related interventions among elderly adults (age≥65) living in the community or in nursing homes in high, middle, and low income countries (Table 1). The outcome measure of interest was vaccine coverage and the exposures of interest were barriers that patients and health services faced to obtain and deliver seasonal influenza vaccine as well as the social health determinants linked to those barriers. Since the aim of our study was not the effectiveness of the vaccine, but the barriers (and their related social determinants) that may affect vaccine uptake, we searched qualitative and descriptive studies that answered questions about structural determinants (e.g. individual socio-economic conditions, public policies, cultural norms) as well as intermediate determinants of health (e.g. attitudes, beliefs, lifestyles) [17,18].

**Table 1:** Inclusion and exclusion criteria

	**Definition of inclusion criteria**	**Exclusion criteria**
Population of interest	Adults ≥65 years old, irrespective of setting	Adults < 65 years old
		Children
		Studies that only include specific subpopulations irrelevant for study aim (i.e. diabetes, HIV)
Intervention of interest	Seasonal influenza vaccination	Interventions not related to seasonal influenza, (AH1N1, pandemic or epidemic periods, vaccine shortage)
	Actions to address barriers against vaccination (i.e. advertising, provider mailings, patient and staff education, visiting nurses)	
Comparisons of interest	Not been immunized	Different vaccines
	Populations without access to immunization	
	Outcome measures of periods before seasonal immunization campaigns	
	Populations or groups without the intervention that promote immunization or avoid barriers	
Exposures of interest	Barriers against vaccination	
	Social determinants of health	
	• Socio-economic & political context: governance, policy (macroeconomic, social, health), cultural and societal norms and values	
	• Social position: education, occupation, income, gender, ethnicity/race	
	• Material circumstances, social cohesion, psychosocial factors, beliefs, behaviours, biological factors	
	• Health care system, distribution of health and well-being	
Outcome of interest	Vaccine coverage	Pharmacological aspects of the vaccine
Study designs	Qualitative (case-studies, ethnography, grounded theory, phenomenology; or specific techniques as focus groups, in-depth interviews, surveys, participant observation)	Reviews, cost-effectiveness or economic analyses
	Quantitative (descriptive, cross sectional, case–control, cohort, randomized controlled trial), mixed-methods	

### Search strategy

**Figure 2:**
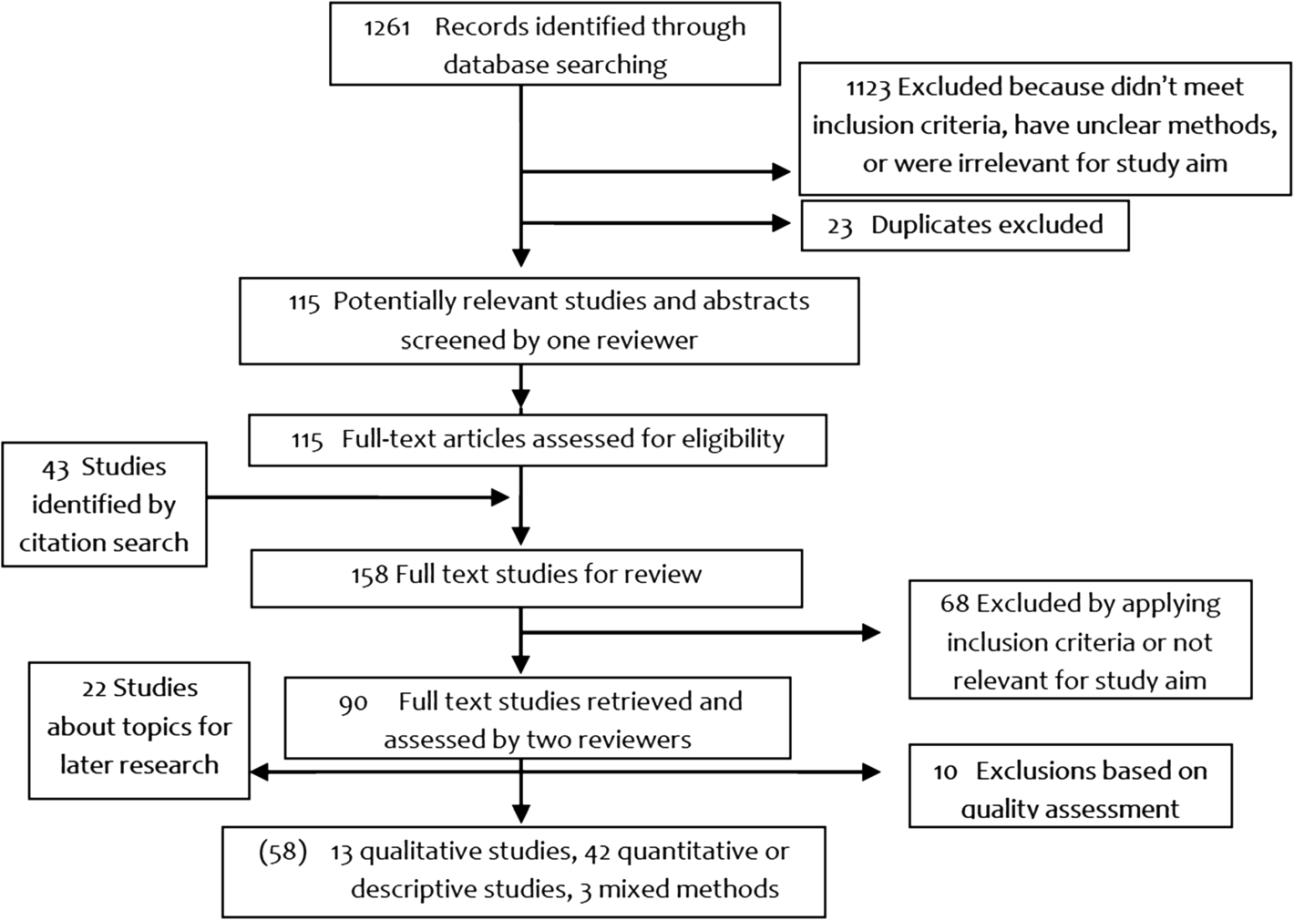
Flowchart of the systematic search.

Following the study protocol, we searched MEDLINE, ISI – Web of Science, PsycINFO, and CINAHL databases. The search strategy considered studies published in the English language between 1980 and 2011, using qualitative research terms or filter terms (Appendix 1). After the first search and before any qualitative screening or data extraction were done, initial research questions were detailed and refined and the search strategy and search terms were modified accordingly. One reviewer (JN) screened all titles and abstracts identified from literature search for relevance based on inclusion criteria (n = 1261) (Table 1). Citations that did not meet the inclusion criteria, were irrelevant for the aim of the study, or had unclear methods were excluded (n = 1123), as were duplicates (n = 23) (Figure 2).

Full paper manuscripts of any titles/abstracts that were considered relevant were obtained (JN) and assessed for inclusion (n = 115) (CF, IH). The reference lists of relevant articles and reviews were back searched for additional studies (n = 43). During full-text review, citations were excluded using the same criteria as above (n = 68). Two authors (CF, IH) assessed the quality and extracted the data of those studies meeting all the inclusion criteria (n = 90).

### Review methods: quality assessment

Two reviewers independently assessed the quality of selected studies by utilizing the Quality Assessment and Review Instrument (QARI) checklist [19]. Data were extracted and compared using adapted forms of the Cochrane Effective Practice and Organization of Care Group (EPOC) which includes separate strategies for qualitative and quantitative data. Each study was read independently by two reviewers (CF, IH). Disagreements between reviewers were resolved by a reconciliation process to achieve consensus. A priori, we decided to appraise the quality of qualitative studies as part of our exploration and interpretation of the paper, but not exclude them based on a rigid checklist, since new insights, grounded in data, might be generated even in studies classified as with low methodological quality. In addition, different structured appraisal approaches may not have consistency in their judgments about the inclusion of studies [20,21].

### Synthesis approach

Our objective in summarizing data from quantitative and qualitative studies was to explore the types and sources of barriers to seasonal influenza vaccination among elderly people and their related social determinants. Since qualitative synthesis was our main interest, we used meta-ethnography [22] and meta-synthesis [23] frameworks, which have been used increasingly in healthcare research [24-26]. For qualitative data synthesis, we created a list of themes and subthemes, compared and juxtaposed them, and determined their relationships using grids and tables [22]. Given the heterogeneity of study designs, we also listed recurrent themes and factors associated with vaccine uptake or refusal in quantitative studies. Thematic analysis was our main approach to joint synthesis and was used to identify major categories, based on primary data rather than prior knowledge [24,26]. Finally, we compared the themes to the Commission for Social Determinants of Health conceptual framework to make them most applicable for policy makers [18].

## Results

Overall, 80 studies were identified as suitable for this review. Given the limited number of studies retrieved about nursing homes (5), homebound patients (2), and interventions (15, from 3 countries) we decided to leave those topics for future studies. From the 58 studies included, 13 used qualitative methods, 3 used mixed qualitative and quantitative approaches and 42 used quantitative or descriptive methods (Appendix 2). There were 13 studies about policy and strategy problems, six about healthcare providers, and 39 regarding patients’ beliefs, attitudes, socio-economic factors, or material circumstances. Nine studies were multinational, including countries from Asia, Europe, Latin America, and the Middle-East. More than half of the studies were done in developed countries. Only six studies included patients from rural areas [27-35]. Qualitative data collection techniques included one-on-one interviewing, questionnaires, key informant selection, focus groups, participant observation, participatory action research, and community mobilization techniques. Quantitative studies encompassed mainly descriptive studies and cross sectional surveys, two ecologic studies, and one controlled trial [36].

**Figure 3:**
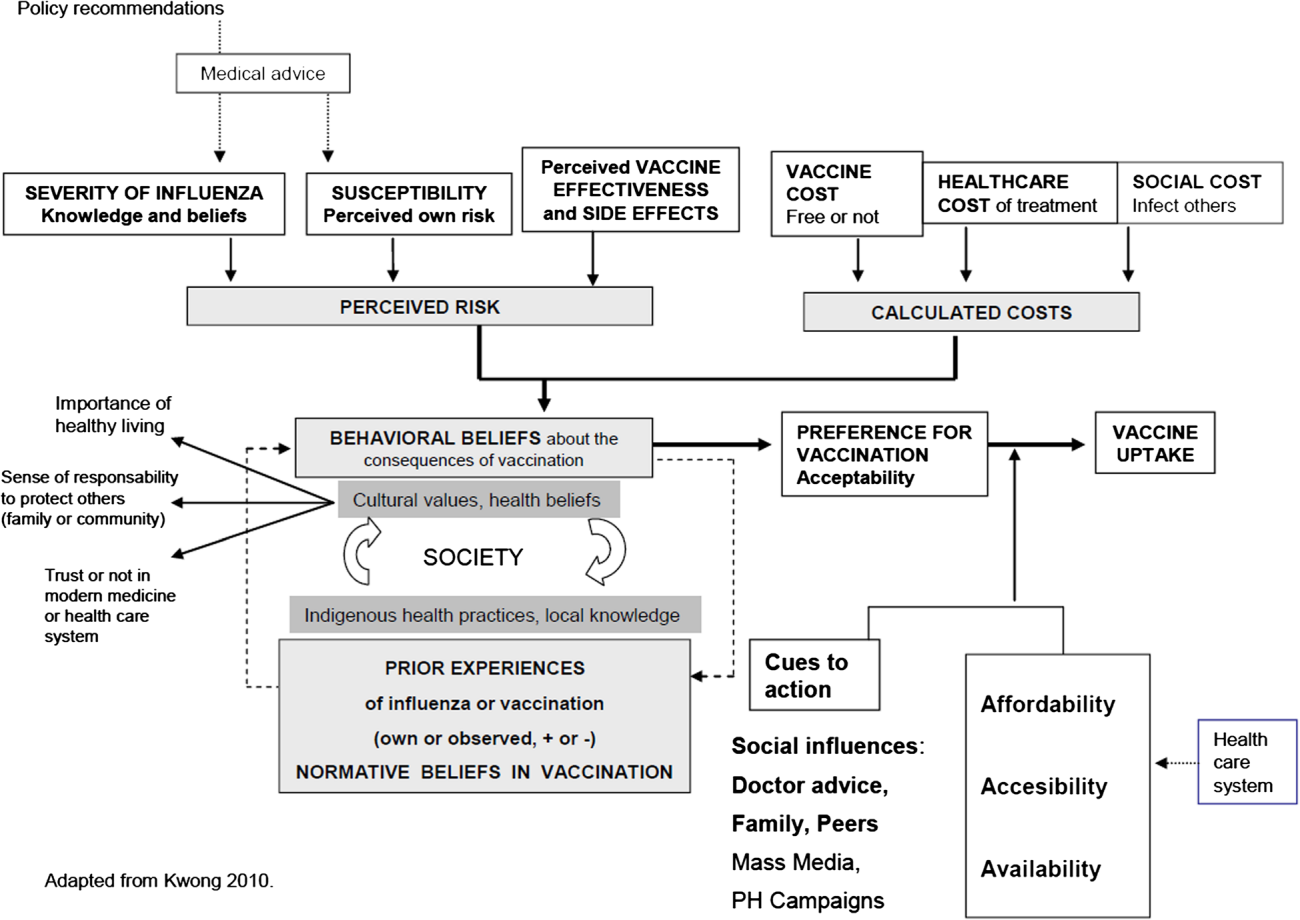
Factors affecting seasonal influenza vaccination in the elderly.

Themes about SDH emerged from each component of the immunization process (policy and governance, healthcare systems, provider, and patient) and were organized according to the categories in the conceptual framework of the SDH (Figure 1): structural, intermediate, and health system determinants (Table 2). In addition, barriers and determinants of patient’s beliefs and behaviors on influenza vaccination are described in Figure 3, while reasons for vaccine acceptance or refusal among elderly adults are summarized in Table 3.

**Table 2:** Themes that emerged at structural, intermediate, and health care systems levels

**Level**	**Theme**	**Summary definition**	**Example citation**	**References**
**1. Structural determinants**				
1.1 Policy and governance level	Vaccine supply	Insufficient seasonal influenza vaccine available for all countries to reduce immunization inequities.		MIV [41], Partridge [38], Kieny [5]
	Finances	Fully funded immunization programs. Reimbursement.	Strong recommendations may be insufficient because patients might accept the vaccine but cannot afford it.	Kunze [43], Lataillade [10], Fedson [39]
	Public health promotion	Awareness of the population through public health information. Promotion about influenza, policy recommendations, and high risk groups for vaccination.	“There was little knowledge about target groups for vaccination in Poland, Turkey and South Africa- countries without immunization programs” (Lataillade).	Lataillade [10], Kwong [44]
			“Awareness of influenza in countries without influenza immunization programs was poor. In South Africa and Turkey in 2005–06 influenza was not distinguished in severity from the common cold” (Lataillade).	
1.2 Provider and healthcare system related	Programmatic barriers: lack of consensus on immunization practices, strategies, and target groups	Recommendations, strategies and practices to vaccinate elderly adults, in outpatient clinics or in nursing homes, are not standardized and vary from country to country. Lack of harmonization of target groups and strategies.		Michel [42], Lataillade [10], Fedson [39], MIV [41], Ropero-Alvarez [37], Kroneman [7], Nakatani [45]
1.3 Patient-related	Gender	Some reports suggest that men are more likely to be vaccinated, and that likelihood of vaccination may change with age for both genders, but both without confirmation in multivariate analyses. No difference by gender was reported in other studies.		Nowalk [47], Shemesh [48], Evans [49], Mangtani [33], Abramson [50], Sarría Santamera [28], Gauthey [51], Chiatti [53], Lopez de Andres [32], O‘Malley [27], Armstrong [52]
	Gender and occupational roles	Occupational roles and responsibilities for childcare may decrease the likelihood of being vaccinated.	“Responsibilities for childcare may also influence access to care” (Adonis).	Adonis [2], Daniels [67], Peña –Rey [32]
			“Non–care givers (of children, old people, or sick people) had an increased probability of being vaccinated” (Peña-Rey).	
	Age	Age has been associated with vaccination uptake in some cross-sectional surveys. Overlap of other factors such as chronic diseases or limitations of functional status must be considered. Others report no difference by age.		Peña-Rey [32], Lopez de Andres [32], O‘Malley [27], Shemesh [48], Evans [49], Chiatti [53], Armstrong [52]
	Marital status	Being married or living with others has been associated with vaccination acceptance in some studies. However, other reports found no relationship. Overlap with issues about social support, access difficulties, or regular preventive health care must be considered.		Mangtagni [34], Damiani [14], O‘Malley [27], Nowalk [47], Gauathey [51], Sarria- Santamera [28], Zimmerman [30] Abramson [50]
	Education	Higher education level has been associated in some reports with higher vaccination rates. However, scales to measure educational attainment and results are not consistent. Others report no influence of education in vaccine uptake or a reverse gradient. Health-related print literacy may have an influence.		Abramson [50], O‘Malley [27], Chiatti [53], Damianni [14], Sarría Santamera [28], Mullahy [55] Bennet [56]
	Race – ethnicity	Higher vaccination rates in whites than in African Americans and Hispanics have been found in the US. Other reports found no differences by ethnic group. In other countries, minority groups may have lower vaccination rates. Language and education may also be related.		O‘Malley [27], Lindley [31], Zimmerman [29,30], Shemesh [48] Nowalk [57], Bardenheier [58]
	Some ethnic groups have specific fears and mistrust of modern medicine, provider, or the health care system	Specific racial groups, like African-American elderly in the US, commented mistrust of the healthcare system and fears.	“We have a general distrust of the medical profession, and we have beliefs in home remedies and that kind of thing.” “Black, people, we have fears. We have fears of the healthcare community - you know, the Tuskegee stuff” (Daniels).	Daniels [67] Harris [63]
	Language and literacy barriers for physician contact or for written campaign information	Minority elders prefer to speak to their provider or read written materials in their native language.	“… I like to go to the Haitian doctor because I can speak with him and do not get embarrassed”. Participants preferred to be interviewed in Creole (Adonis-Rizzo).	Adonis-Rizzo [2], Lasser [71], Daniels [67], O‘Malley [27]
	Socio-economic level	This multi-dimensional concept was measured with different variables across studies. Lower socio-economic status has been correlated with lower vaccination uptake; however, other reports showed no difference, or even reverse gradient.		Peña- Rey [32], O’Malley [27], Nowalk [47], Chiatti [53], Mangtani [34], Sarría-Santamera [28], López de Andres [32].
	Presence of chronic diseases	Vaccination rates have been higher among patients with chronic diseases, adjusting for gender, age, and other factors. Other reports have found the association only in bivariate analysis.		Chiatti [53], Evans [49], Damiani [14], López de Andres [32], O‘Malley [27], Abramson [50]
	Cultural values and health beliefs: healthy living for prevention of illness	Health beliefs include the desire to improve health, the importance of healthy living, and behaviors and lifestyles important for prevention of illness and health promotion. Refusers may have reliance on healthy lifestyle and avoidance of close contacts. Community perceptions to not get vaccinated are a barrier to immunization.	“My father had a saying, which he repeated again and again: it is better to pay the butcher and the baker than the doctor … I still continue today in the way my father and mother brought us up, meat, fish, vegetables …” (acceptor) “keep away from people, you know, because I think myself, what gives you flu if you don’t have the needle, I think you get flu by being with a lot of people you see” (acceptor who became refuser) (Telford)	Sengupta [65], Telford [66], Cornford [62], Adonis-Rizzo [2], Evans [64], Daniels [67], Kwong [69], Zimmerman [30]
			“Well, it’s for my health so I’m going to do it, if it’s the best thing for me” “If they (churchgoers) felt that the flu shot were to their advantage, they would stand in line for five or 10 minutes and get the shot and then go home” (Daniels).	
	Sense of community - protect others	Sense of responsibility to protect self and others in the community. Word-of-mouth from the community to get the flu shot.	“I think so, yes I think so a lot (friends say that to you as well, a general feeling that vaccination is a good thing to do), that’s the first think on their minds. You all see that you all get it. It reminds you and you remind others. Make sure we all get it” (Vaccinated from UK).	Sengupta [65], Schensul [36]
	Local beliefs, perceptions, and knowledge: indigenous health practices to avoid or treat influenza	Local understandings of the cause of influenza as a natural illness (as opposed to other that come from outside- competing paradigms), that can be treated with natural remedies, food and warm clothing, as well as the awareness of the potential severity of the disease.	“I thought that it is just a big cold … Can you explain it to me? … I don’t know what is it, I though I was just a cold” [they consider flu a natural illness, traditional preventive practices would seem sufficient, and the potential for complications was not considered] (Adonis-Rizzo).	Kwong [8,69], Cameron [70], Sengupta [64] Adonis-Rizzo [2], Daniels [67]
	Trust or lack of it in the health care system, provider, or modern medicine	Lack of trust in the vaccine. Fears and mistrust in the healthcare system.	“Well I would say, if you get recommendations from the Government and the medical profession and they both urge you to do these things, well do’em …” (acceptor) “The first time, I had it on the Tuesday morning and by night I was out with my sisters and friends, .. I went shivering, shaking, so I left them, and got a taxi home and took a couple of powders and went to bed. The next morning I was as right as rain, and I’ve had it twice since then and it’s never affected me”. “We have a general distrust of the medical profession, and we have beliefs in home remedies and that kind of thing.” (Daniels). “I don’t like doctors and hospitals all that much” (Evans)	Harris [63], Telford [66], Evans [64], Daniels [67]
**2. Intermediate Determinants**				
2.1. Policy and governance level	Housing – place of residence	Data about differences between rural and urban settings are contradictory and depend on country and health system characteristics. Place of residence may determine ease of access to vaccination, and socio-economic status may affect living conditions (central heating or not, rented or owned house).		Mangtani [33,34], Lopez de Andres [32], O‘Malley [27], Sarría Santamera [28], Zimmerman [30]
2.2. Provider and healthcare system related	Type of practice	VA system has higher vaccination rates compared to non-VA practices due to its use of multimodal interventions to increase rates such as freestanding vaccination clinics, patient reminders, standing orders, and regular assessment of vaccination rates with incentives to clinicians.		Zimmerman [30]
	Influenza vaccination in the previous year	One of the most important predictors of vaccine uptake. However, does not always reflect current attitudes towards vaccination.		Lasser [71], Harris [63], Telford [66], Kwong [8], Evans [49], Zimmerman [30], Armstrong [52] Cornford [62], Nowalk [47]
2.3. Patient-related	Behavioural beliefs about consequences of vaccine uptake	Different frameworks proposed. Behavioral beliefs are based on the patient’s probability calculation of susceptibility to and severity of influenza, their knowledge about vaccine effectiveness, and their healthcare and social cost of the vaccine.		Bosompra [35], Zimmerman [29], Nowalk [47], Kwong [8,44]
	Social influences. Advice from family or peers may trigger vaccine acceptance	Cautious willingness. Patients trust their family members, as well as peers or known community members’ advice.	“My daughter told me about it, I had it done based on her recommendation. I had it done because I trust my daughter. I didn’t hesitate (Vaccinated, South Korea, Kwong). “I have to ask my children before that. If they say I should, I’ll receive this injection. If they say no, I will not receive it …” “I will talk with my friends, people of the same age and with the same health condition could help us decide whether to get the flu shot or not. If they decide against it, I do not want to do it either” (Payaprom).	Evans [64], Adonis-Rizzo [2], Lasser [71], Payaprom [46], Schensul [36], Daniels [67], Kwong [8,61], Zimmerman [29,30], O‘Malley [27], Lau [73]
	Prior experiences of influenza or with influenza vaccine (IV)	Own or observed prior experiences, positive or negative, of influenza or with IV in previous years.	“My brother in law got it and he was in the hospital for more than a month with the flu, with fever, vomits, he got everything.” “Ay cuñada don’t do it”, so I never got it. No, no, I won’t do it” (Lesser).	Lasser [71], Harris [63], Telford [66], Kwong [8,44], Bardenheier [58] , Bosompra [35], Evans [64]
	Concerns about the vaccine safety, effectiveness, side effects. Fear of pain, injections, and getting the disease with the vaccine	Negative experiences or anecdotes and fear of mild or severe side effects and pain. Refusers are more likely to believe IV had serious side effects, that it is ineffective, and be skeptical or have no confidence in the vaccine.	“… it was purely that I didn’t like needles and people, you’d hear about these side-effects; al the side affects you have from that flu jab, oh you can’t lift your arm and you’re sick” (refuser who became acceptor) (Telford) “I’ve heard so many people being bad (ill) after it …” (Evans)	Lasser [71], Telford [66], Evans [64], Daniels [67] Kwong [44,61], Armstrong [52], Bardenheier [58], Harris [63], Cameron [70], Sengupta [65], Adonis –Rizzo [2], Shemesh [48], Zimmerman [29,30], Kwong [44]
			“My sister has the flu every year, and she takes the shot! … I said well that doesn’t make sense. And she has it real bad. So I never bothered with it”. “I thought if I took the flu shot I might get a cold, get the flu”. “I take it, the flu shot, then, I get the flu” (Cameron) “I don’t think it gives you overall protection” (refuser).	
	Willingness to continue, adherence	If positive experiences occurred after the first shot, every year the patient will continue with IV.	“I took it and I will take it from now on” (Schensul).	Harris [63], Schensul [36], Lau [73], Zimmerman [30]
			“The habit of being vaccinated” (Zimmerman).	
	Perceived risk or susceptibility	Perceived susceptibility based on patient’s awareness and previous knowledge of the disease.	“I think the good part outweighs the risky part of it. Just like with normal shots, the same thing. You may get pneumonia you may get sick, but probably 96% of people, this is going to save them in some way”, “I take it so I won’t be as sick. It does make me feel bad, but I still get up and go. You know?”	Evans [49], Kwong [8,44], Telford [66], Bosompra [35]
	Perceived severity of influenza, previous awareness and knowledge about influenza	Fear of disease. Knowledge and beliefs about severity of influenza and its contagiousness. Some patients think influenza is serious for others, but not themselves.	Contrasting opinions. “It is not the worst thing in the world. It can be dealt with” vs. “You really, really, really feel really sick”, “you feel like you going to die” (Cameron). Being knowledgeable about the severity of the flu: “I have had the flu, and I know how sick you can get from it” (Payaprom).	Cornford [62], Cameron [70], Payaprom [46], Kwong [8,44], Bardenheier [58]
	Lack of knowledge. Misconceptions about influenza or IV. Curative *vs.* preventive effect. Misconceptions about adult vaccination	Misconceptions about influenza and about the vaccine might be prevalent in some communities, and should be approached with correct information sources.	“I normally get the tetanus booster every 10 years as it comes up. And I can see the benefits of the pneumonia, the pneumovac … For older people. As far as the flu, I’ve never had the flu, so I don’t get the vaccine” (Daniels). “The vaccine is good, really, so that it will take out all the infection that you have, like that, really”, “then they put the vaccine (flu), the flu comes, and you throw out a lot of phlegm” (Daniels).	Payaprom [46], Daniels [67], Armstrong [52]
	Perceived or self-appraised health status. Awareness of IV indications	Self-perception of “poor” health has been associated with vaccine acceptance, whereas self- perception of “good” health may be a reason for non-uptake. Perceived risk (low or high, age related) of contracting influenza, the knowledge of personal risk factors and awareness of vaccine indications are important factors. Some elders believe that influenza carries no risk for healthy older people. Some patients are not able to relate the potential risk of mortality from influenza to themselves or to others unless a pre-existing condition or other health issue is present.	“Young people can fight it”, “I think because you’re older, resistance is low” (Cameron). “Well, I really don’t be sick … I ’m in a pretty good shape the doctors say”. “I am a person that don’t catch colds very easily” (Cameron) “Yes, I believe it could be (that death could occur) but not directly because of the flu, because when the flu is developed where there is high temperature and low defenses, that could trigger another kind of disease that is present but unseen. … he could have lived for a long time, but he caught a very bad flu, and it forced him to stay in bed. That happens to elderly people when they stay in bed for a long time, especially on their backs, and it gets complicated. It complicates with the lung, and he died, but truly, you can’t say that it was only because of the flu” (Daniels).	Cornford [62] Evans [49], Cameron [70], Sengupta [65], Payaprom [46] Daniels [67], Kwong [69], Zimmerman [30], Damiani [14], Peña-Rey [32], López de Andres [32], Mangtani [33].
	Perceived cost of the vaccine. Free, low, or high cost. Reimbursements	If financial barriers exist, even patients who have accepted the vaccine will not receive it. Misunderstandings or misinformation about cost of influenza vaccine exist.	“Right now, I don’t have a good income and so even if I wanted to couldn’t get the flu shot” (Kwong) “I got mine because it was free”, “some of the problem [is] they don’t have health insurance and cannot afford to pay for these things” “Also cost, better health insurance for, insurance making it (flu vaccine) available that way” (Sengupta).	Kwong [8,61], Cameron [70], Sengupta [65], Lasser [71], Lau [73]
	Perception of health care cost and social cost	Cost of treatment of disease or its complications.	“The flu shot is not expensive if it is effective $80 is cheap … I need to pay several hundreds to treat influenza-related diseases. The cost of the flu shot is lower” (vaccinated from China, Kwong).	Kwong [8], Lau [73], Cameron [70], Sengupta [65], Lasser [71]
		An increase of the chances to infect their family members, particularly for elderly adults living with other persons including family and grandchildren.		
			My doctor advised my daughter to have me get the flu shot, so I do so every year. My daughter as two lovely children … and they usually get sick during winter … We live together” (vaccinated from Greece, Kwong).	
**3. Health systems**				
3.1. Policy and governance level	Accessibility to seasonal influenza vaccine	Different aspects of accessibility for the elderly are distance to the health center, convenience of its location, transportation, language, access to healthcare, and legal status.		
	Convenience. Vaccine delivery may be enhanced in more convenient places for elderly people	Elderly people may consider having the vaccination if it is provided locally, near their home, or in convenient community delivery places as pharmacies, shopping malls, and supermarkets.	“The health center is fine. It’s near our houses and it’s not crowded. If it’s the hospital, you have to spend one day because the hospital service is very slow and my children have to take me there” (Payaprom). “In terms of flu shots, it’s, I think, a whole matter of convenience” “The only reason my husband had a flu shot was that he happened to be in supermarket, and they were doing them” (Daniels).	Payaprom [46], Cameron [70], Daniels [67], Adonis –Rizzo [2],
	Faith based organizations or other community organizations as venues for adult immunization delivery	Churches were perceived as convenient and accessible community locations, trusted organizations in the community, and sites where a significant number of older adults regularly convened. Peer models, bulletin posters, and support from faith-based leaders may be used to give encouragement.	“I think the church is a good place for vaccinations because a lot of people go there”, “I think it would be a good place. Obviously, there needs to be other places too, for people that don’t go to church. But I think you would find a lot of them - I know that the older generation does tend to go to church or go back to the church at some point” (Daniels).	Daniels [67]
	Depend on others for transportation	Most patients did not drive and are dependent on their children, friends, or church members for transportation. This dependence makes return visits more difficult to schedule.	“It is hard to drive … If my children can take me where the vaccine is being offered, I will definitely take it” (Adonis)	Adonis-Rizzo [2], Daniels [67], Zimmerman [29], Lasser [71]
	Language and literacy barriers for physician contact or written campaign information	Cultural competencies of provider are desirable and needed to deliver preventive messages and to convince patients to get vaccinated.	“… I like to go to the Haitian doctor because I can speak with him and do not get embarrassed”. Participants preferred to be interviewed in Creole (Adonis).	Adonis-Rizzo [2], Lasser [71], Daniels [67], O‘Malley [27], Kwong [61]
	Immigration status	Having to sign forms with names and addresses may elicit fears related to legal immigration status. Some adults fear losing access to services.	“Yes, yes, I have heard commentaries that they don’t get near the vaccines because “I am illegal”. Now, yes and they are distrustful, really, because you have to sign papers with your name” “The hospital that I go, they tell you every year to go and get the vaccine. Then, I go and get the vaccine because I am scared that they would take away my assistance” (Daniels).	Daniels [67]
	Affordability and cost	Elderly people may consider having influenza vaccination if it is provided free of charge. Some patients felt that knowledge of the costs and benefits of the IV may be a motivating factor to increase immunization.	“They should (get vaccination). But what would they do? Elderly people without any income support can only live day by day” (Payaprom) “ If the black community were more aware of these free vaccines - I mean, it’s going to be cost-effective for them health wise, and also for HMOs because you don’t need to fill up a hospital with a bunch of people with pneumonia” (Daniels).	Payaprom [46], Cameron [70], Daniels [67], Lau [73], Kwong [8,61], Sengupta [65], Lasser [71],
	Health insurance or preventive services. Lack of knowledge about insurance coverage and IV cost	Some patients had limited knowledge and understanding of the existing healthcare insurance coverage for the flu vaccine. Lack of health insurance, insurance status, and cost were important considerations.	“The lack of care would make people to seldom receive the vaccines or prevent them from receiving them. It is that they don’t have a doctor, people don’t have access to doctors “.	Adonis-Rizzo [2], Lasser [71], Daniels [67], Zimmerman [29,30], O‘Malley [27]
	Recent visits to the health care center	The use of medical care or services and the frequency of contacts with the health care system may increase the opportunities for receiving counseling and immunization (but not always).		Abramson [50], O’Malley [27], Peña-Rey [32], Evans [49]. Sarría-Santamera [28]
3.2. Provider and healthcare related	Health professionals’ influences. Advice from physician or professional health care provider	Physicians’ recommendations are one of the most frequently reported influences on immunization status. Patients trust their provider’s advice. However, providers cover many topics in visits and may not talk about IV or recommend it. Providers must be proactive, have consistency in their recommendations, and promote vaccination with patient reminders.	“My doctor never told me about it .... If he recommended I would take it” (Adonis). “I don’t remember being reminded to get a flu shot. I used to go to a general practitioner and perhaps he could mention that … My gynecologist doesn’t talk about flu shots. I have an oncologist, he doesn’t talk about flu shots - so most of my doctors are more linked to specific conditions, they’re specialist, and they don’t talk about flu shots”. “But, I don’t remember on any regular basis- any doctor or nurse- saying to me … for instance; I just got notice in the mail that it was time to have my mammogram. And then I thought - okay. I will do that. But I’ve never gotten anything in the mail or from my doctors saying “It is time to have your flu shot”.	Adonis-Rizzo [2], Armstrong [52], Bardenheier [58], Evans [49,64], Lasser [71], Daniels [67], Kwong [8,61], Gauthey [51], Zimmerman [29,30], Lau [73], Payaprom [46], Schensul [36], Shemesh [48], O’Malley [27], Nowalk [47] , Sengupta [65], Müller [74]
	Physicians’ awareness, knowledge, attitudes, and practice. Communication strategies and cultural competence	Healthcare provider acceptance of seasonal influenza vaccine depends on demographic factors such as years of practice, being up-to-date with scientific journals, and cultural factors. Other factors that promote vaccine acceptance are communication strategies include information giving skills, cultural competency, empathy, persistence, trust, and vaccination by the provider. Availability and distribution of the vaccine in a timely basis are determinants of the perceptions of the practitioners. Other environmental factors include logistical and competing demands.		Zimmerman [31], Pavia [76], Lasser [71], Pyrzanowski [75], O’Malley [27]
3.2. Patient related	Sources of information	Suggestions on how to effectively provide information on the significance of influenza immunization to the health of older adults. Suggested strategies included those through the healthcare system, media, community-based organizations, and churches. Advertisements through television, radio, newspaper and magazines may not be as effective as desired.	“I think if you had multiple sources of information - if you had it through the church, the announcements at church or the bulletin, on TV, on the radio, in the newspapers … then you could remember where and when (to get the flu shots)”. (Daniels)	Daniels [67], Zimmerman [29]
			Source of information about influenza vaccine recommendations are medical professionals, television, newspapers, friends/ family and other (Zimmerman 2002)	

**Table 3:** Reasons for vaccine acceptance or refusal among elderly adults

**Reasons for acceptance**	**Reasons for refusal**
**Personal facilitators**	**Personal barriers**
Positive attitude to prevention. Desire to avoid flu	Not knowledgeable about flu or severity of flu
Recommended by physician or nurse, by mail, or personally	Low educational attainment, low health-related printed literacy
Recommended by friend or relative	Misconceptions about immunization in adults
Being knowledgeable about the severity of the flu	Perception of low susceptibility to the disease due to good health status: “Not likely to contract influenza”
Having “not very good health”	Lack of knowledge about indications of influenza vaccine in general or for himself
Having a chronic condition that puts them at higher risk for getting the flu	Unjustified medical reasons (allergy to drugs, chronic illness)
Having had a job that puts them at a higher risk for getting the flu	Lack of interest, forgot to get it, never considered it before
Positive prior experiences with the vaccine or only mild side effects	Anecdotes or prior negative experiences with the vaccine
Benefits of the flu shot	Concerns or fears about risks of the flu shot
Prevention	Side effects, pain, fear of injections
Decreases symptom severity of the flu	Fear of undisclosed shot contents
Greater ability to do day-to-day activities	Misconception of getting influenza from the vaccine
“Do not pass influenza to my family and friends”	Disbelief in vaccine efficacy
Perceived consequences: thought that unvaccinated persons would probably contract influenza	Previous bad reactions and past problems with flu shot batches
Free vaccine: Social Security pays for it	Accessibility problems: transport, distance, convenience, unable to attend because ill health, language
Getting older	Cost: patient unable to afford the shot. Lack of knowledge about insurance coverage
**Structural facilitators**	**Structural barriers**
Physician’s recommendation/reminder to get the shot	Word of mouth from the community to not get a flu shot
Visits to the physician	Irregular or lack of preventive healthcare
Word-of-mouth from the community to get a flu shot	Doctor did not recommended the vaccine, missed opportunities
Written or visual media promoting flu shot use	Lack of access
Vaccine is free or low cost	Regarding vaccine shortage
Type of physician	Regarding location access

### Structural determinants

Beyond individual factors influencing exposure and vulnerability, the conceptual framework on SDH emphasizes the role of structural determinants, which are the social and political mechanisms that generate and maintain social hierarchy and stratification such as labor markets, education systems, social protection regimes, political institutions, and cultural and societal values. These factors influence the level of power and resources that a society can redistribute among its different population groups. These structural mechanisms shape social hierarchies according to income, education, occupation, social class, gender and race/ethnicity. They are the root cause of inequalities in health.

### Policy and governance level

An initial consideration is that most reports included in this review focused in high-income countries. Low and middle-income countries (as well as rural and peri-urban populations) were underrepresented, demonstrating the larger structural determinants that influence availability, access, and information about benefits of seasonal influenza vaccination in the elderly. The main themes identified in this review about policies and programs regarding seasonal influenza immunization and socially explained immunologic inequities were: insufficient seasonal influenza vaccine (SIV) availability for all countries, need of fully funded immunization programs and public health promotion, and lack of consensus on immunization practices and harmonization of target groups (Table 2).

#### Insufficient seasonal influenza vaccine available for all countries to reduce immunization inequities

While there has been an increase in the availability of SIV since 2006 by increased production, there are still gaps in the availability of influenza vaccine production capacity for low and middle-income countries in the event of a pandemic [5,37,38]. The continuous need to update antigen selection on a yearly basis according to the circulation of viral strains is a major health system barrier leading to inequities in the availability of the vaccine in non-producing countries, particularly low and middle income countries. While there has been an expansion in the number of countries using seasonal influenza vaccine, today, no country has fully implemented its own vaccine recommendations. Substantial variations in influenza vaccination coverage persists among countries in most regions of the world [9,37,39-41].

### Provider and healthcare system

#### Programmatic barriers: lack of consensus on immunization practices, strategies, and target groups

As important as the availability of a yearly SIV is the design and implementation of programmatic strategies and activities to achieve adequate levels of effective immunization coverage among high-risk populations (including adults ≥65 years). Even in industrialized countries, significant population groups at risk of complications from influenza are not vaccinated or refuse the vaccine. Countries that provide reimbursements for healthcare practitioners to administer SIV or that provide the vaccine within public health insurance coverage tend to have higher seasonal influenza vaccination rates for the elderly [7,10,39,41-45].

Currently, there is little agreement on ideal vaccination strategies and on the selection of target groups to receive SIV. There is also lack of consensus of the benefit of seasonal influenza vaccination in the elderly [40]. In some regions, the lack of harmonization of vaccination strategies and of selection of high-risk populations has contributed to insufficient vaccination coverage of some target groups [9,42]. Some studies have shown that nationwide vaccination programs maybe a better strategy to decrease influenza-associated mortality in the elderly [45,46].

### Patients

Determinants of health related to personal characteristics have been studied to understand which factors may increase the likelihood of vaccine uptake or to identify the population groups to which intervention efforts must be focused on. However, the findings have not always been consistent, and some of the reasons that could explain the variability are: 1) associations found in cross-sectional surveys are not causal associations and confounding might affect them; 2) some demographic variables (such as education, socio-economic status, or household income) are defined in different ways or proxy measures are used, thus limiting the comparability among studies and countries; 3) real differences may exist among groups, regions, or countries due to characteristics of the population, the influenza program, or the healthcare system.

#### Gender

Some studies have found that men are more likely to be vaccinated in comparison with women [34,47-49]. However, such differences were present only in bivariate analysis and disappeared in multivariate regression analysis (i.e. 85% of men vaccinated vs. 75% of women [33]; 76.1% of men vs. 60.3% of women, OR 2.1 p < 0.0001) [50]. Sarría-Santamera reports that with increasing age, the likelihood of vaccination among women decreased, but the likelihood among men increased [28]. Gauthey found that although vaccine coverage was higher among men (39.9 vs. 32.7%, p = 0.04), differences between genders became smaller and statistically insignificant with increased age [51]. Other reports have found no difference by gender [27,52-54].

#### Age

Age has been associated with vaccination uptake in different cross-sectional surveys. Chiatti found that influenza vaccination was more likely among adults ≥85 years (73.5%, adjusted odds ratio (AOR) 1.8, 95% CI 1.6-2.0, p < 0.01), or those 75–84 years old (70.5%, AOR 1.7, 95% CI1.6-1.8, p < 0.01), than in those aged 65–74 [53]. Similarly, Lopez de Andres found a greater vaccination likelihood in patients ≥75 years (OR 2.4, 95% CI 2.0-2.8), and 70–74 years (OR 1.6, 95% CI 1.3-2.0), than in those aged 65–69 years [54]. The effect of age on vaccination may appear among patients with or without associated chronic diseases [53,54]. However, limited functional status may decrease the likelihood of vaccine uptake since access might depend on transportation or assistance, unless living in a nursing home. One study found no relationship between age and influenza vaccination status [52].

#### Marital status

Being married and having social support has been associated with vaccination acceptance. Abramson reports higher vaccination rates in married patients compared to unmarried patients (74% vs. 56%, AOR 2.1, 95% CI 1.3-3.5, p = 0.003), though weaker associations have been also reported (AOR 1.45, 95% CI 1.05-2.01 [32]; AOR 1.3, 95% CI 1.2-1.4), [27]. In Italy, widowed and single persons were less likely to be vaccinated compared to married persons (OR 0.83, 95% CI 0.77-0.88) [14]. People who live alone with limited assistance may have less access, irregular preventive health visits, and less support from family members. However, other reports have found no relationship of marital status with vaccine uptake [28,51]. One study reported higher immunization rates among single or never married patients (93%, AOR 9.2, 95% CI 2.9-29, p = 0.001), compared to married (84%, AOR 2.6, 95% CI 1.3-5.4, p = 0.01), widowed (80%, AOR 2.0, 95% CI 1.0-3.9, p = 0.05), or separated/divorced (69%) persons [30].

#### Education

Higher education levels have been associated with higher vaccination rates; however, different scales have been used to measure education attainment, limiting the comparability among the studies. Abramson found the association strongest in bivariate analysis (55% with ≤8 years, vs. 72% with ≥9 years of school, p = 0.0007) [50], though a slight positive gradient with increased educational level was also reported (AOR for ≥college degree 1.3, 95% CI 1.2-1.5) using multivariate regression [27]. Conversely, a reverse gradient was reported in Italy, with greater vaccination rates in the group without a primary school degree (66.5%) than in the group with a high school degree or more (59.3%), although this difference was not significant in multivariate analysis [53]. Other studies have found no influence of education on vaccination [14,28]. Individuals with higher educational attainment may have more access to regular preventive healthcare, resources to overcome barriers, awareness about healthy lifestyles, and confidence to ask the physician directly about the vaccine [55]. Conversely, elderly persons with lower educational attainment may have lower socio-economic status, may be more likely to have strong cultural beliefs, and may rely more heavily on indigenous health practices. Furthermore, health-related print literacy (the ability to use health information from printed sources to make appropriate health decisions) significantly mediates racial and education-related disparities in self-rated health status and use of influenza vaccination [56]. Therefore, different levels of educational attainment are related to health literacy and influenza vaccination use.

#### Race-ethnicity

Ethnicity has been recognized as one of the markers of health inequity and most studies on race come from the US where social and health inequalities among ethnic groups have been well-documented [57]. For example, in a cross-sectional survey of Medicare beneficiaries in the US, white patients had significantly higher odds of influenza vaccination compared to black patients (AOR 1.5, 95% CI 1.4-1.7) adjusting for patient, physician, health system, and area level characteristics [27]. A wide difference in the proportion of African Americans (50.2%), Hispanics (31.7%), and whites (20.7%) that had not received an influenza vaccination has also been reported [58]. In a survey of inner-city neighborhood health centers, Zimmerman reported lower vaccination rates for blacks (60%) in comparison with white patients (79%) [29]. In other countries there are few reports about vaccination rates in ethnic minority groups. In a survey conducted in a health fair in Israel, there was higher influenza vaccine coverage among Arabic (80.8%) and Hebrew (68.7%) speakers, as compared with Russian (33.5%) speakers, immigrants from the former Soviet Union [48]. Conversely, other reports have found no significant differences by race [30], particularly after controlling for socio-demographic characteristics [59]. Health literacy significantly mediates racial/ethnic disparities in vaccination uptake [56].

#### Socio-economic status (SES)

SES is a multi-dimensional concept that may vary with context. SES was measured as income, occupation, highest individual class within the household, or deprivation index for the district in which patients resided. Lower SES has been found to be correlated with lower vaccination uptake [32,34,47]. Patients with higher income were found to have significantly higher probabilities of vaccine uptake in Spain (AOR 1.4, 95% CI 1.01-1.9) and in the US (AOR 1.3, 95% CI 1.1-1.5) [27,32]. Interestingly, a reverse gradient has been found in some countries where health policies and influenza programs have been effectively implemented. In Italy, influenza vaccination was more prevalent in lower social classes (65.1%, AOR 1.2, 95% CI 1.1, 1.3, p < 0.01), than in upper social classes (56.9%) [53]. In Brazil, in an ecologic study comparing age-specific (≥65 years) mortality rates before and after the onset of yearly vaccination, deprived areas of the city (with poorer profiles of human development, lower health indices, and lower incomes) had more significant decreases in mortality by pneumonia and influenza during the vaccination period [60]. Other surveys found no association of SES with vaccination status [28,54].

#### Presence of chronic diseases (CD)

Because CD are an indication for influenza vaccine administration, the frequency of vaccination is expected to be higher with the presence of CD [61]. For example, in Italy vaccination was significantly higher among patients with severe CD such as severe diabetes, cardiac disease, or chronic pulmonary obstructive disease (70.7%, OR 2.0, 1.8-2.1, p < 0.01); and mild CD (60.1%, OR 1.4, 1.3-1.6, p < 0.01); than in their absence (47%) adjusting for gender, age, social class, education, self-reported health, and having a general practitioner visit in last month [53]. In the UK, the likelihood of vaccination increased as the number of CD increased, adjusted by gender, age, health status, and hospital visits (for 1 CD, AOR 2.5, 95% CI 1.9-3.2; for 2 CD, AOR 3.2 95% CI 2.0-5.1; and for ≥3 CD, AOR 4.0. 95% CI 1.2-12.9) [49]. Having at least one CD was associated with a higher vaccination rate in Italy (AOR 1.53, 95% CI 1.45-1.62) [14], in Spain (AOR 1.6, 95% CI 1.3-1.9) [54], and in the US with two or more CD vs. none or one (AOR 1.6, 95% CI 1.4-1.7) [27]. In Israel, 75.2% of patients with CD were vaccinated in comparison to 64.7% without CD (p = 0.0067), but the difference was statistically significant only in the bivariate analysis [50].

#### Cultural values and health beliefs about vaccination

Vaccinated persons are more confident in the effectiveness of the vaccine and value its benefits to their families and communities. For some elderly people, health represented the absence of medical diseases and the ability to be independent and actively engage with other people [62]. Protecting and helping others, ideals of “healthy living,” and trust in providers may be part of cultural norms [30,36,49,62-69]. The contribution of vaccination to family and community health may be stressed in countries with a strong cultural heritage that values the protection of the family. In these communities, patients may give more weight to the social benefit of vaccination than to the financial cost [8]. Conversely, unvaccinated patients are more likely to trust indigenous health practices, rely on healthy lifestyles, and doubt the effectiveness of vaccines. Local understandings of the causes of influenza place it as a natural illness, which can be treated with broths and warm clothing [8,65,68-70]. Therefore, effective interventions to promote influenza vaccination must understand cultural beliefs and practices and use them to complement immunization [8].

### Intermediate determinants

We grouped in this category factors that operated at the individual level including lifestyles, personal beliefs, perceptions, behaviors, individual choices material conditions, or psychosocial factors. By adopting a social causation of disease approach, we find that the unequal distribution of these factors becomes the primary mechanism through which socio-economic position generates health inequities [18].

### Policy and governance level

#### Housing – place of residence

As a person’s place of residence may determine ease of access to vaccination, some studies have included an index of deprivation of the area of residence, or categorized areas as urban or rural [34]. Simultaneously, SES influences living and health conditions and perceptions related to them. For instance, patients living without central heating or living in rented accommodation were 10% less likely to be vaccinated compared to patients with central heating or home-owners. Practices located in areas with high indices of deprivation or with relatively high mortality rates reported lower vaccine uptake in adults >74 years [33,34]. In Spain, living in a town with more than 10,000 inhabitants increased the likelihood of patient’s vaccination (AOR 1.4, 95% CI 1.2-1.6) [54]. In contrast, one report showed that urban settings had a lower likelihood of influenza vaccination than rural towns (AOR 0.7, 95%CI 0.6-0.8), while another study demonstrated that people 65–69 years old living in cities with more than one million inhabitants had lower vaccination rates than those living in cities with less than one million inhabitants [27,28].

### Provider and healthcare system

#### Type of practice

Zimmerman reports (without statistical significance) higher vaccination rates in patients from Veterans Affairs (VA) practices (91%) compared with non-VA practices in inner-city (67%), suburban (79%), or rural (79%) locations [30]. Higher vaccination rates in the VA system may be due to its use of multimodal interventions to increase rates such as freestanding vaccination clinics, patient reminders, standing orders, and regular assessment of vaccination rates with incentives to clinicians [30].

#### Influenza vaccination in the previous year

Quantitative studies have also found that vaccination in the prior year is one of the most important predictors of vaccine uptake in the elderly. For instance, Zimmerman reports that in a sample of 1,007 telephone interviews, 99% of respondents who stated they were vaccinated planned on being vaccinated in the following year, compared to only 25% of respondents who stated they were not currently vaccinated (p < 0.001) [30]. Nowalk presents similar findings in a study in which 98% of respondents vaccinated in the 2000–2001 influenza season reported their intention to obtain an influenza vaccination in the following year, compared to 39% of those who were unvaccinated (p = 0.0001) [47]. Therefore, intention is one of the strongest predictors of behavior. If patients have a positive initial vaccination experience, they are likely to seek the vaccination year after year and get “in the habit” of being vaccinated [30,36]. However, one qualitative study found that vaccination status of previous year did not always reflect current attitudes towards vaccination, as a few vaccinated patients decided they would not be vaccinated the following year [62].

### Patient

#### Behavioral beliefs about consequences of vaccine uptake

Behaviors related to immunization have been analyzed in different frameworks [8,29,35,47,69]. One such model posits that patients’ vaccination preferences are determined by behavioral beliefs based on their probability calculation of susceptibility to influenza and their utility calculation of vaccine, healthcare, and social costs [8]. Behavioral beliefs are dependent on their normative beliefs that are also moderated by structural determinants such as cultural values and health beliefs (Figure 3).

#### Prior experiences of influenza or vaccination

A patient’s own or observed prior experiences of influenza or influenza immunization are strong forces guiding behavior preference and normative beliefs [30,35,52,64,71]. The prevalence of beliefs in favor of vaccination was dependent on how predominantly the belief became normative in the country.

#### Concerns about the vaccine

Patients in different countries refuse the vaccine because they think the vaccine itself can cause illness, is ineffective, has moderate/severe side effects, produces pain, or contains undisclosed ingredients. Conversely, vaccinated people had confidence in the vaccine effectiveness and recognized the vaccine as a preventive measure which may decrease the severity of the symptoms and likelihood of contagion to family and households (Table 3).

#### Perceived risk or susceptibility

This concept refers to the self-estimated risk that patients calculate depending on their awareness about the severity of influenza, the indications of the vaccine, the probability of contagion, and their susceptibility. Older age or having a chronic condition which increases their risk for influenza may make patients realize their susceptibility. Being knowledgeable about the severity of the flu and the benefits of the vaccine are facilitators for vaccination [58,62,65,70,72]. Perceived risk is also shaped by confidence in vaccine effectiveness, fear of side effects, and fear of getting the flu with the vaccine [29,30,48,52,61,64,66-69,71].

Societal and cultural differences should be considered in terms of perceived risk and susceptibility. In China, some elderly people believed in the benefits of vaccination, had no cost barriers, and did not fear side effects. However, if they did not perceive themselves as susceptible, they were not afraid of getting influenza and did not believe its complications to be serious [69]. In another study specifically among never immunized people aged 65 and over, predictors of acceptance of SIV were the perceived likelihood of getting influenza (AOR 2.1, 95% CI 1.1-4.0, p = 0.03), the recognition that side effects of IV were less risky than the disease itself (AOR 4.9, 95% CI 2.3-10.8, p < 0.001), and the recognition that everyone over 65 years should receive the vaccine (AOR 76.5, 95% CI 16.1-363.8, p < 0.001) [49].

#### Perceived or self-appraised health status

Patients who perceive themselves with poor health status are expected to have higher vaccination rates if they believe they have a higher susceptibility of contracting influenza or suffering its consequences (Table 2). Assessing self-health status as “poor” or “no good” was associated with higher vaccine use in Spain (AOR 1.2, 95% CI 1.1-1.5) [32,54] and in Italy (AOR 1.5, 95% CI 1.4-1.6) [14]. Similarly, patients with perceived “good” health were significantly less likely to be vaccinated (50.3%, AOR 0.73, 95% CI 0.68-0.76, p < 0.01) in comparison with patients with “fair” health status (64.5%, as the referent), or with “bad” health status (71.1%) [53]. In another study, patients refusing SIV who reported good health (44%) were likely to have better SES (owner occupied housing with central heating), live in non-urban areas, and have no previous experiences with influenza [33]. Other reports showed no association of health status with vaccination uptake [28,50].

#### Calculated cost of vaccination

Patients calculate the expected cost or utility when assessing their own risk and experiences with influenza vaccination. The calculation is compounded by: 1) the cost of the vaccine itself (critical barrier); 2) the healthcare costs for the treatment of influenza, or its complications if the patient remains unvaccinated and get the disease; and 3) the social cost, which is the perceived risk of infecting family members or caregivers [8,65,70,71,73,74].

#### Health systems

The health system is a social determinant of health and its role becomes particularly relevant through the issue of access, which determines who will be able to get a healthcare intervention. Health systems can address differences in exposure and vulnerability by improving equitable access to care and promoting policies that tackle bottlenecks such as geographic barriers to access healthcare. Although we consider the overall the health system as an intermediary determinant, given its important role, we grouped all factors related to it in this section.

### Policy and governance level

#### Accessibility of seasonal influenza vaccine

Accessibility is an important concern for elderly adults and has multiple aspects: distance and convenience of health center locations, hours of immunization services, transportation, language and literacy, health insurance, and legal status. Vaccine delivery may be enhanced in convenient locations for elderly people, such as pharmacies, supermarkets, and churches (Table 2). Elderly people may be dependent on others for transportation [68] and ease of access through different means of transportation has been reported [29]. Language is another access barrier as elders from minority groups prefer to speak to their provider in their native language [68]. Some measures to provide information and increase the likelihood of vaccine acceptance are the production of diffusion materials in the patient’s native language and conducting information meetings in their language or with a facilitator [36]. Literacy barriers must be also considered, since specific populations may have low literacy levels or no schooling and may ignore written information [61]. Thus, to avoid language and cultural barriers, cultural competency is one of the strategies to improve communication with patients and to convince them to accept immunization [71].

#### Affordability of seasonal influenza vaccine

Cost is an important determinant in countries where patients have to pay for the vaccine [69]. Elderly people may consider having the influenza vaccination if it is provided free of charge. Some patients reported limited knowledge and understanding of the existing healthcare or insurance coverage for the flu vaccine [29,30,67,70,72,73], particularly among those that had irregular or no access to preventive healthcare [27,65,67,68,71].

#### Recent visits to the healthcare center

A positive association between the frequency of visits to a physician and influenza immunization might be expected since patients can receive advice or the immunization itself. For instance, in Israel 72% of subjects who had visited their physician in the last three months were vaccinated, in comparison to 55% among those without recent visits (AOR 2.6, 95% CI 1.5-4.8, p = 0.0006) [50]. In the US, four or more outpatient visits during the year previous to the survey increased the likelihood of influenza vaccination (AOR 1.6, 95% CI 1.5-1.8) [27]. Similarly, Spanish women with at least one physician visit in the last two years had significantly higher probabilities of being vaccinated (AOR 4.8, 95% CI 2.6-8.9) [32]. Sarría-Santamera reported that when the time of last visit was greater than six months, the likelihood of not being vaccinated increased with age (65–69 years: AOR 1.9, 1.1-3.3; ≥70 years: AOR 2.3, 95% CI 1.5-3.6) [28]. However, there was a significant association between outpatient or inpatient hospital visits during the previous year and reported influenza vaccine uptake in multivariate analyses [49].

### Provider and healthcare

#### Health professionals’ influences

Several studies have found that physician advice is significantly associated with vaccination uptake [30,47,48,51,71,73,74]. Patients trust their physicians and also follow advice from trusted family members and peers [8,29,36,61,64,68,72]. There are reports that many physicians do not offer the vaccine to their patients. Potential explanations may be that doctors cover many topics during the visits, give low priority to vaccination in adults, forget to propose it, underestimate the key influence vaccines can play, disbelieve vaccine effectiveness, believe that patients will refuse it, or believe the vaccine is not convenient and easily accessible during the visit [8,36,49,58,72]. Other studies have suggested that some patients make appointments specifically to get vaccinated [8]. In addition, receiving a reminder from a doctor to get the flu shot is an important structural facilitator for immunization [65].

#### Availability and physicians’ awareness, knowledge, attitudes, and practice

An important determinant of influenza vaccination is the perception of community-based health practitioners about adequacy of vaccine stocks such as the availability and distribution of the vaccine on a timely basis and interruptions in its supply during some seasons [31,75]. Physicians’ awareness and agreement with official recommendations for vaccination were consistently associated with higher immunization status. In particular, proactive office systems (with standing orders tracking, chart checklist, vaccine clinics), education, and physicians may influence patients’ intentions to receive seasonal influenza vaccine [31]. In Italy, a survey among general practitioners found that a positive attitude towards hospitalizations being reduced by SIV was significantly more common in physicians with fewer years of professional activity (p = 0.05), who work more hours per week (p = 0.013), and who relied on scientific journals as a source of information (p = 0.002) [76]. Moreover, a qualitative study about encounters between primary care physicians and elderly patients found that communication strategies and information giving skills, such as sharing of power and responsibility, empathy, and treating the patient like a person, facilitated communication and promoted acceptance of flu vaccination. Other facilitators included cultural competence, provider introduction of the discussion, persistence throughout the visit, trust and rapport among patient and physician, and provider vaccination of the patient [27,71].

### Patient

#### Sources of information about vaccine

As noted earlier, the recommendation of influenza vaccination by the physician, family, and peers can motivate vaccine uptake [27,29,30,51,52,61,73,74]. Vaccinated patients, compared to unvaccinated patients, were more likely to report that their doctor (99% vs. 80%, p < 0.001) and family/friends (90% vs. 59%, p = 0.007) thought they should get the SIV [47]. Important sources of information for elderly people are newspapers, television, magazines, radio, and media in general [27,30]. However, few surveys asked if the information given through national influenza campaigns were seen or were considered useful to promote vaccination. For instance, being exposed to advertisements arguing the need for SIV via television, radio, magazines, or newspapers was not significantly associated with vaccine uptake [49,61].

## Discussion

The effectiveness of influenza immunization for both seasonal and pandemic influenza depends upon a timely and sufficient supply of the vaccine. Even if all social barriers to implement or strengthen seasonal influenza immunization in the elderly are removed, equitable access to SIV remains an issue for many low- or middle-income countries. There is an increasing push by the World Health Organization to eliminate disparities in seasonal immunization rates among and within Member States as part of resolution World Health Assembly (WHA) 56.19, which in 2003 recommended the adoption and/or strengthening of influenza vaccination policies to increase seasonal influenza vaccination coverage among populations at high risk of complications and death [5]. The WHO Global Influenza Vaccine Action Plan has improved the availability of seasonal influenza vaccine for a significant number of middle and low-income countries [37,38]. As a result, influenza vaccination is increasing throughout the world, especially in middle-income countries of Latin America and Central and Eastern Europe. Of note, countries that provide reimbursement for healthcare practitioners to administer influenza vaccine or provide seasonal influenza vaccine within their public health insurance coverage tend to have higher seasonal influenza vaccination for the elderly [39,41]. However, no country has fully implemented its own vaccine recommendations and substantial variations in influenza vaccination persist among countries in most regions of the world [37,39,40]. Even in wealthy industrialized countries, significant population groups at risk of complications from influenza remain unvaccinated or refuse the vaccine. In this systematic review, we identified the importance of social determinants of health in regards to seasonal influenza immunization. This is relevant given the current efforts to expand seasonal influenza vaccination into low and middle income countries. Decision makers, when designing public health interventions, can consider the full range of determinants that influence the effective coverage of programs. As many of these determinants operate outside the health sector, decision makers will also need to consider the adoption of mechanisms for intersectoral action.

Socio-cultural aspects and social support may affect vaccine acceptance. At an individual level, factors such as physicians’ advice, cost, convenience, perceived susceptibility, prior experiences, health status, personal beliefs, and misconceptions about the vaccine and the disease mainly shaped the vaccine acceptance among this population [30,47,48,51].

In contrast to other routine immunizations, the effectiveness of seasonal influenza vaccine requires yearly administration to high-risk groups that develop complications or death associated with influenza infection. Due to the constant risk of antigenic drift, there is a need for yearly selection of circulating viral strains, and the effectiveness is directly related to the degree of match between vaccine virus and circulating strains. This feature adds other important financial and programmatic barriers to the expansion of seasonal immunization agendas that has burdened some national immunization programs in prioritizing financial and human resources.

Once the historical, financial, political, and epidemiological dimensions of implementing a seasonal influenza immunization program are achieved [39], there are operational dimensions of seasonal influenza vaccine programs to consider. As important as availability of a yearly seasonal influenza vaccine is the design and implementation of a variety of strategies and activities to achieve adequate levels of effective immunization coverage among high-risk populations. Even in high-income countries with established SIV programs targeting high-risk populations and available vaccine, the rates of vaccination are far from ideal, while elderly adults at risk of influenza remain reluctant of vaccination [77]. Currently, there is little agreement on the ideal vaccination strategies and the ideal selection of target groups to receive seasonal influenza vaccine [40,78]. For instance, there is even lack of consensus on the benefit of seasonal influenza vaccination in the elderly [40]. Recent Cochrane Reviews [12] have cast doubt on the scientific evidence behind current consensus recommendations to vaccinate the elderly against seasonal influenza. Moreover, in some regions, a lack of harmonization of vaccination strategies and selection of high-risk populations have contributed to insufficient vaccination coverage of some target groups [42].

Differences in vaccination strategies have provided varied results. In Japan, vaccination against influenza among school-aged children demonstrated an important impact in the elderly [79], while other studies have shown that nationwide vaccination programs may be better alternatives to decrease influenza-associated mortality in the elderly [46]. On the other hand, non-specific preventive measures such as hand-washing, distance, and wearing a mask during periods of risk are useful [40] and even desirable given the difficulty to distinguish influenza from influenza-like illness and the concurrent circulation of diverse respiratory viruses. These supplemental and basic measures should be emphasized in prevention messages, especially when and where there are no means to avoid the barriers against influenza vaccine uptake.

The significance of qualitative research has been increasingly recognized in health sciences disciplines; however, efforts to integrate or synthesize qualitative findings have been relatively limited, particularly with the topic of influenza vaccination [23,24]. Evidence from qualitative and quantitative studies that examine social determinants of health, factors that shape the delivery and implementation of interventions, and the experience of persons involved in providing and receiving interventions improves the scope and relevance of systematic reviews to policy-makers and practitioners [20,80,81]. Furthermore, situating the results of the review within the social determinants of health model may provide a conceptual framework particularly useful for global policy making [18,82].

This study has certain limitations. The study may have source bias, since we did not search in the grey literature or unpublished studies and used qualitative research and filter terms instead of free text searching [24]. We did not search specific regional databases that may be more suitable to report research from developing countries. As only studies written in English were included, we may have missed studies from developing countries published in other languages. Since more than half of the studies were conducted in developed countries and in urban areas, low and middle-income as well as rural regions may be underrepresented. The inclusion of studies relying on cross-sectional survey methods may introduce selection bias as it is not possible to control for individuals refusing to be interviewed. Most importantly, associations between vaccine uptake and other variables found in cross-sectional surveys do not involve causal relationships [32], and such associations may be confounded by other factors [40,78,83]. Limitations of the meta-synthesis methodology similarly include the inability to infer causal relationships from mixed qualitative and quantitative data. There is little consensus on the use of quality appraisal in qualitative meta-synthesis [20]; therefore, we did not exclude any qualitative studies based on quality rating schemes. However, our search strategy was appropriate to explore social determinants in our population of interest.

The fact that most studies evaluated in this systematic review came from high-income countries illustrates that seasonal influenza vaccine is not routinely offered in low and middle-income countries. Particularly, we can assume that non-served and underserved populations are not routinely offered seasonal influenza vaccine because this is not a standard public health practice or a public health priority in many low-and medium income countries, where determinants such as vaccine availability as well as financial and political barriers prevent the effective deployment of these interventions. Finally, because influenza vaccine effectiveness may be suboptimal, especially in older people [12], the opportunity to prevent influenza related complications in this population will benefit from the development of more immunogenic vaccines that could be used and shared at affordable costs to populations in high-, middle-, and low income countries. Newer influenza vaccines such as the universal influenza vaccines may potentially change the landscape of influenza vaccine protection by providing long term protection and avoiding the need of yearly revaccination [5].

## Conclusion

Seasonal influenza remains a public health challenge with important economic and social tolls. While the precise epidemiology has not been completely deciphered in middle-and low-income countries, it is likely that this burden is shared globally. Our results also highlight that policies, practices, and vaccination strategies against seasonal influenza vaccination are influenced by social determinants where vaccine is routinely available. Some of these determinants are health-system related, provider-related, or patient-related resulting in variable coverage levels within countries. While vaccination efforts continue to expand to middle-and low income countries, there is minimal representation of underserved populations in currently available reports. This issue demonstrates that larger social determinants influence the availability and vaccination practices in these areas. Incorporating a framework that takes into account social determinants of health into vaccine policy design and implementation may foster immunization equity among the most vulnerable populations against seasonal influenza and likely other vaccine preventable diseases. Incorporating a social determinants framework will also allow decision makers to identify where determinants are located (within or outside the health sector) and serve to adopt mechanisms for intersectoral action to address those determinants originating outside the realm of the health sector.

## Appendix 1

Keywords used for search

#1 Seasonal influenza vaccine: “Influenza Vaccines”[MeSH] OR “Influenza, Human/prevention and control”[MeSH], “seasonal influenza vaccine” , “seasonal influenza vaccines”, NOT pandemic, NOT epidemic.

#2 Elderly adults: (old* or pension* or retire* or aged or elderly or senior* or geriatric*) or (“long-term care” or “nursing care” or “palliative care” or “homes for the aged” or “nursing homes”), or (Community dwelling, homebound, community ).

#3 Qualitative research: (Qualitative Research[MeSH]) OR (Nursing Methodology Research[MeSH]) OR qualitative or ethnograph* or phenomenol* or ethnonurs* or grounded theory* or (lived experience*) or narrative* or (life experiences) or (cluster sample) or (action research) or (observational method) or (content analysis) or (thematic analysis) or (constant comparative method) (discourse analysis) or (focus group*) or (ethnological research) or ethnomethodolog* or (mixed methods).

#4 SDH, inequalities: “Healthcare Disparities”[Mesh] OR “healthcare disparities” OR “health care disparities” OR “Healthcare Disparity” OR “Health care Disparity” OR “Health Status Disparities”[Mesh] OR “health status disparities” or “health status disparity” OR “Social Class” [Mesh] OR “social mobilities” OR “ social mobility” OR “Poverty Areas”[Mesh] OR “poverty areas” OR “poverty area” OR “slums” OR “slum” OR “ghetto” OR “ghettos” OR “Educational Status”[Mesh] OR “educational status” OR “Educational Achievement*” OR “Illiteracy” OR “Literacy” OR “Cross-Cultural Comparison”[Mesh] OR “Cross-Cultural Comparison” OR “Cross-Cultural Comparisons” OR “Transcultural Studies” OR “Transcultural Study” OR “Prejudice” [Mesh] OR “Prejudice” OR “Prejudices” OR “Racism” OR “Social Discrimination” OR “Sexism” OR “Gender Bias” OR “Sex Bias” OR “Sex Discrimination” OR “Ageism” OR “Segregation” OR “caste” OR “castes” OR “ resource poor” OR “inequities” OR “inequality” OR “inequalities” OR “Socioeconomic Factors”[Mesh] OR “Socioeconomic Status” [Mesh] OR “social class” OR “social classes” OR “socioeconomic status” OR “Social Environment”[Mesh] OR “social environment” OR “social environments” OR “Social Conditions”[Mesh] OR “social conditions” OR “social condition” OR “low income populations” OR “low income population” OR “Vulnerable populations” [Mesh] OR “vulnerable populations” OR “vulnerable population” OR “Sensitive Populations “ OR “Sensitive Population” OR “Disadvantaged” [TI] OR “Social Determinants”[TIAB] OR “socio-economic status” OR “Policy” OR “Government”, OR “social health determinants”, OR “low income”, OR “minority”, poverty,

#5 Barriers:

Barriers, barrier, access barriers, travel time, geographic location, availability of services, geographic distribution of services, provider location, acceptability barriers, acceptance barrier, health behavior, knowledge, belief, beliefs, health belief, attitude, attitudes, mistrust, fear, adequate funding, user fees, out of pocket payment, co-payment, subsidy, referral, service referral, diagnostic kits, surveillance, vaccine stocks, sustainable stocking, immunization barriers.

#6 Interventions:

Advertising, campaign, provider mailings, standing orders, registry-based telephone calls, telephone calls, remainder systems, patient education, staff education, visiting nurses, outreach, visiting physician, patient satisfaction, multi- level, multi-level intervention, single intervention, intervention, interventions, immunization program, program, multidisciplinary, preventive health services, community participation.

Search strategy

#1 Seasonal influenza

#2 Elderly

#3 Qualitative

#4 SDH

#5 Barriers

#6 Interventions

#7 #1 AND #2 Elderly SIV

#8 #4 OR #5 SDH and barriers

#9 #7 AND #8 Elderly, SIV, SDH, Barriers

#10 #3 OR #6 Qualitative research or potential interventions

#11 #9 AND #10 SIV, elderly, barriers, SDH, interventions

## Appendix 2

Studies for Data Abstraction

1. Abramson ZH, Cohen-Naor V. Factors associated with performance of influenza immunization among the elderly. Isr Med Assoc J 2000; 2: 902–7.

2. Adonis –Rizzo MT, Jett KF. Health beliefs of Haitian elders related to influenza prevention. Public Health Nurs 2006; 24(1): 18–25.

3. Armstrong K, Berlin M, Schwartz S, Propert K, Ubel PA. Barriers to influenza immunization in a low-income urban population. Am J Prev Med 2001; 20(1): 21–5.

4. Bardenheier BH, Wortley PM, Winston CA, Washington ML, Lindley MC, Sapsis K. Do patterns of knowledge and attitudes exist among unvaccinated seniors? Am J Health Behav 2006; 30 (6): 675–83.

5. Barnes GJ, Quigley C. Flu vaccination in nursing homes: a survey of nursing home managers. J Public Health 2006; 28(1): 56–60.

6. Bennett IM, Chen J, Soroui, White S. The contribution of health literacy to disparities in self-rated health status and preventive health behaviors in older adults. Ann Fam Med 2009; 7: 204–11.

7. Bosompra K, Ashikaga T, Ruby A. Attitudes, perceived norms and intentions: A needs assessment study of the influenza immunization intentions of elderly citizens in Vermont. J Rural Health 2004; 20(2): 125–30.

8. Cameron KA, Rintamaki LS, Kamanda-Kosseh M, Noskin GA, Baker DW, Makoul G. Using theoretical constructs to identify key issues for targeted message design: African American seniors’ perceptions about influenza and influenza vaccination. Health Commun 2009; 24: 316–26.

9. Chiatti C, Di Rosa M, Barbadoro P, Lamura G, Di Stanislao F, Prospero E. Socioeconomic determinants of influenza vaccination among older adults in Italy. Prev Med 2010; 51: 332–3.

10. Cornford CS, Morgan M. Elderly people’s beliefs about influenza vaccination. Br J Gen Pract 1999; 49: 281–4.

11. Damiani G , Federico B, Visca M, Agostini F, Ricciardi W. The impact of socioeconomic level on influenza vaccination among Italian adults and elderly: A cross-sectional study. Prev Med 2007; 45: 373–9.

12. Daniels NA, Juarbe T, Rangel-Lugo M, Moreno-John G, Pérez-Stable EJ. Focus group interviews on racial and ethnic attitudes regarding adult vaccinations. J Natl Med Assoc 2004; 96(11): 1455–61.

13. Evans MR, Watson PA. Why do older people not get immunized against influenza? A community survey. Vaccine 2003; 21: 2421–7.

14. Evans MR, Prout H, Prior L, Tapper-Jones LM, Butler CC. A qualitative study of lay beliefs about influenza immunization in older people. Br J Gen Pract 2007; 57: 352–8.

15. Fedson DS, Hannoun C, Leesex J, Sgrenge MJW, Hampson AW, Bro-Jarrgensen K, et al. Influenza vaccination in 18 developed countries 1980–1992. Vaccine 1995; 13(7): 623–627.

16. Ferreira Antunes JL, Alves Waldman E, Borrell C, Paiva TM. Effectiveness of influenza vaccination and its impact on health inequalities. Int J Epidemiol 2007; 36: 1319–26.

17. Gauthey L, Toscani L, Chamot E, Larequi T, Robert CF. Influenza vaccination coverage in the geriatric population of the State of Geneva, Switzerland. Eur J Public Health 1999; 9: 36–40.

18. Harris LM, Chin NP, Fiscella K, Humiston S. Barrier to pneumococcal and influenza vaccinations in black elderly communities: mistrust. J Natl Med Assoc 2006; 98(10): 1678–84.

19. Jansen A, Sanders EA, Nicho KL, van Loon AM, Hoes AW, Hak E. Decline in influenza-associated mortality among Dutch elderly following the introduction of a nationwide vaccination program. Vaccine 2008; 26: 5567–74.

20. Jefferson T, Di Pietrantonj C, Debalini MG, Rivetti A, Demicheli V. Inactivated influenza vaccines: Methods, policies, and politics. Journal of Clinical Epidemiology 2009; 62: 677–686.

21. Kroneman M, Paget WJ, van Essen GA. Influenza vaccination in Europe: an inventory of strategies to reach target populations and optimize vaccination uptake. Euro-surveillance 2003; 8:6.

22. Kunze U, Groman E, Böhm G, Kunze M. Influenza vaccination in Austria, 1982–2003. Wien Med Wochenschr 2007; 157:98–101.

23. Kwong JC, Stukel TA, Lim J, McGeer AJ, Upshur REG, et al. The effect of universal influenza immunization on mortality and health care use. PLoS Med 2008; 5(10): e211.

24. Kwong EW, Lam IO. Chinese older people in Hong Kong: health beliefs about influenza vaccination. Nurs Older People 2008; 20(7): 29–33.

25. Kwong EW, Lam IO, Chan TM. What factors affect influenza vaccine uptake among community-dwelling older Chinese people in Hong Kong general outpatient clinics? J Clin Nurs 2009; 18: 960–71.

26. Kwong EW, Pang SM, Choi P, Wong TK. Influenza vaccine preference and uptake among older people in nine countries. J Adv Nurs 2010; 66(10): 2297–308.

27. Lataillade C, Auvergne S, Delannoy I. 2005 and 2006 seasonal influenza vaccination covarage rates in 10 countries in Africa, Asia Pacific, Europe, Latin America and the Middle East. J Public Health Policy 2009; 30: 83–101.

28. Lasser KE, Kelly B, Maier J, Murillo J, Hoover S, Isenberg K, et al. Discussions about preventive services: a qualitative study. BMC Fam Pract 2008; 9: 49.

29. Lau JTF, Kim JH, Yang X, Tsui HY. Cross-sectional and longitudinal factors predicting influenza vaccination in Hong Kong Chinese elderly aged 65 and above. J Infect 2008; 56: 460–8.

30. Lau L, Ying L, Lau YH. Prevalence and correlates of influenza vaccination among non-institutionalized elderly people: An exploratory cross-sectional survey. Int J Nurs Studies 2009; 46: 768–77.

31. Lindley MC, Groom AV, Wortley PM, Euler GL. Status of influenza and pneumococcal vaccination among older American Indians and Alaska Natives. Am J Public Health 2008; 98 (5): 932–8.

32. Lopez de Andres A, Carrasco P, Hernández-Barrera V, Vázquez-Fernández S, Gil A, Jiménez-García R. Influenza vaccination among the elderly Spanish population: trend from 1993 to 2003 and vaccination-related factors. Eur J Public Health 2006; 17(3): 272–7.

33. Macroepidemiology of influenza vaccination MIV study group. The macro-epidemiology of influenza vaccination in 56 countries, 1997–2003. Vaccine 2005; 23: 5133–43.

34. Mangtani P, Breeze E, Stirling S, Hanciles S, Kovats S, Fletcher A. Cross-sectional survey of older peoples’ views related to influenza vaccine uptake. BMC Public Health 2006; 6:249.

35. Mangtani P, Breeze E, Kovats S, Edmon SW, Roberts JA, Fletcher A. Inequalities in influenza vaccine uptake among people aged over 74 years in Britain. Prev Med 2005; 41:545–53.

36. Manuel DG, Henry B, Hockin J, Naus M. Health behavior associated with influenza vaccination among healthcare workers in long-term-care facilities. Infec Control Hosp Epidemiol 2002; 23(10): 609–14.

37. Michel JP, Lang PO, Baeyens JP. Flu vaccination policy in old adults: Need for harmonization of national public health recommendations throughout Europe. Vaccine 2009; 27: 182–183.

38. Mullahy J. It’ll only hurt a second? Microeconomic determinants of who gets flu shots. Health Econ 1999; 8: 9–24.

39. Müller D, Szucs TD. Influenza vaccination coverage rates in 5 European countries: A population based cross-sectional analysis of the seasons 02–03, 03/03 and 04/05. Infection 2007; 35: 308–19.

40. Nakatani H, Sano T, Iuchi T. Development of a vaccination policy in Japan: current issues and policy directions. Jpn J Infect Dis 2002; 55:101–11.

41. Nowalk MP, Zimmerman Rk, Shen S, Jewell IK, Raymund M. Barriers to pneumococcal and influenza vaccination in older community-dwelling adults (2000–2001). J Am Geriatr Soc 2004; 52: 25–30.

42. Nowalk MP, Tabbarah M, Terry MA, Raymund M, Wilson SA, Fox DE, Zimmerman RK. Using quantitative and qualitative approaches to understand racial disparities in adult vaccination. J Natl Med Assoc 2009;101(10): 1052–60.

43. O’Malley AS, Forrest CB. Immunization disparities in older Americans. Determinants and future research needs. Am J Prev Med 2006; 31 (2):150–8.

44. Partridge J, Kieny MP, World Health Organization H1N1 influenza vaccine Task Force. Global production of seasonal and pandemic (H1N1) influenza vaccines in 2009–2010 and comparison with previous estimates and global action plan targets. Vaccine 2010; 28(30): 4709–12.

45. Pavia M, Foresta MR, Cargone V, Angelillo IF. Influenza and pneumococcal immunization in the elderly: knowledge, attitudes, and practices among general practitioners in Italy. Public Health 2003; 117: 202–7.

46. Payaprom Y, Bennet P, Burnard P, Alabaster E, Tantipong H. Understandings of influenza and influenza vaccination among high-risk urban dwelling Thai adults: a qualitative study. J Public Health 2009; 32(1): 26–31.

47. Peña-Rey I, Pérez-Fainós N, Sarría-Santamera A. Factors associated with influenza vaccination among elderly Spanish women. Public Health 2004; 118: 582–7.

48. Pyrzanowski JL, Daley MF, Crane LA, Barrow J, Babbel C, Kempe A. A qualitative study of physicians’ experiences ordering and receiving influenza vaccine during the 2005–2006 influenza season. Prev Med 2008; 47: 225–8.

49. Ropero-Álvarez AM, Kurtis JH, Danovaro-Holliday C, Ruiz-Matus C, Andrus JK. Expansion of seasonal influenza vaccination in the Americas. BMC Public Health 2009; 9: 361.

50. Sarría-Santamera A, Timoner J. Influenza vaccination in old adults in Spain. Eur J Public Health 2003; 13:133–7.

51. Schensul JJ, Radda K, Coman E, Vazquez E. Multi-level intervention to prevent influenza infections in older low Income and minority adults. Am J Community Psychol 2009; 43:313–29.

52. Sengupta S, Corbie-Smith G, Thrasher A, Strauss RP. African American elders’ perceptions of the influenza vaccine in Durham, North Carolina. N C Med J 2004; 65(4): 194–9.

53. Shemesh AA, Rasooly I, Horowitz P, Lemberger J, Ben-Moshe Y, Kachal J, et al. Health behaviors and their determinants in multiethnic, active Israeli seniors. Arch Gerontol Geriatr 2008; 47: 63–77.

54. Telford R, Rogers A. What influences elderly peoples’ decisions about whether to accept the influenza vaccination? A qualitative study. Health Educ Res 2003; 18(6): 743–53.

55. vanEssen GA, Palache AM, Forleo E, Fedson DS. Influenza vaccination in 2000: Recommendations and vaccine use in 50 developed and rapidly developing countries Vaccine 2003:21;1780–5.

56. Zimmerman RK, Mieczkowski TA, Wilson SA. Immunization rates and beliefs among elderly patients of inner-city neighborhoods health centers. Health Promot Pract 2002; 3: 197–206.

57. Zimmerman RK, Santibanez TA, Janosky JE, Fine MJ, Raymund M, Wilson SA, Bardela IJ, Medsger AR, Nowalk MP. What affects influenza vaccination rates among older patients? An analysis from inner-city, suburban, rural, and veterans affairs practices. Am J Med 2003; 114:31–8.

58. Zimmerman RK, Nowalk MP, Bardella IJ, et al. Physician and practice factors related to influenza vaccination among the elderly. Am J Prev Med 2004; 26:1–10.

## Abbreviations

IV: Influenza vaccine; SES: Socio-economic status; SIV: Seasonal influenza vaccine; VPD: Vaccine preventable diseases; WHO: World Health Organization.

## Competing interests

The authors declared that they have no competing interests.

## Authors’ contributions

JN prepared the review protocol, performed the systematic search, prepared the manuscript, and finalized the submission. IH conducted the systematic review data abstraction, prepared the manuscript, and edited the manuscript. AS, DA, and CV conceived of the study, participated in its design, and helped to draft the manuscript. CF conducted the systematic review data abstraction, prepared the manuscript, and edited the manuscript. All authors read and approved the final manuscript. AS, DA, and CV are staff members at WHO. The authors alone are responsible for the views expressed in this publication and they do not necessarily represent the decisions, policy, or views of WHO.

## Pre-publication history

The pre-publication history for this paper can be accessed here:

http://www.biomedcentral.com/1471-2458/13/388/prepub
